# Fire and Plant Diversification in Mediterranean-Climate Regions

**DOI:** 10.3389/fpls.2018.00851

**Published:** 2018-07-03

**Authors:** Philip W. Rundel, Mary T. K. Arroyo, Richard M. Cowling, Jon E. Keeley, Byron B. Lamont, Juli G. Pausas, Pablo Vargas

**Affiliations:** ^1^Department of Ecology and Evolutionary Biology and Institute of the Environment and Sustainability, University of California, Los Angeles, Los Angeles, CA, United States; ^2^Department of Ecological Science, Faculty of Sciences, Institute of Ecology and Biodiversity, University of Chile, Santiago, Chile; ^3^African Centre for Coastal Palaeoscience, Nelson Mandela University, Port Elizabeth, South Africa; ^4^Sequoia Field Station, Western Ecological Research Center, United States Geological Survey, Reston, VA, United States; ^5^School of Molecular and Life Sciences, Curtin University, Perth, WA, Australia; ^6^Centro de Investigaciones sobre Desertificación, University of Valencia, CSIC, Valencia, Spain; ^7^Department of Biodiversity and Conservation, Royal Botanical Garden of Madrid, CSIC, Madrid, Spain

**Keywords:** Mediterranean-type climate, speciation, species diversity, Cape Region, southwestern Australia, California, Mediterranean Basin, central Chile

## Abstract

Despite decades of broad interest in global patterns of biodiversity, little attention has been given to understanding the remarkable levels of plant diversity present in the world’s five Mediterranean-type climate (MTC) regions, all of which are considered to be biodiversity hotspots. Comprising the Mediterranean Basin, California, central Chile, the Cape Region of South Africa, and southwestern Australia, these regions share the unusual climatic regime of mild wet winters and warm dry summers. Despite their small extent, covering only about 2.2% of world land area, these regions are home to approximately one-sixth of the world vascular plant flora. The onset of MTCs in the middle Miocene brought summer drought, a novel climatic condition, but also a regime of recurrent fire. Fire has been a significant agent of selection in assembling the modern floras of four of the five MTC regions, with central Chile an exception following the uplift of the Andes in the middle Miocene. Selection for persistence in a fire-prone environment as a key causal factor for species diversification in MTC regions has been under-appreciated or ignored. Mechanisms for fire-driven speciation are diverse and may include both directional (novel traits) and stabilizing selection (retained traits) for appropriate morphological and life-history traits. Both museum and nursery hypotheses have important relevance in explaining the extant species richness of the MTC floras, with fire as a strong stimulant for diversification in a manner distinct from other temperate floras. Spatial and temporal niche separation across topographic, climatic and edaphic gradients has occurred in all five regions. The Mediterranean Basin, California, and central Chile are seen as nurseries for strong but not spectacular rates of Neogene diversification, while the older landscapes of southwestern Australia and the Cape Region show significant components of both Paleogene and younger Neogene speciation in their diversity. Low rates of extinction suggesting a long association with fire more than high rates of speciation have been key to the extant levels of species richness.

## Introduction

Scholars have long sought explanations for patterns in global and regional species richness. Primary among studies of this type has been the quest to explain latitudinal patterns in species diversity, with diversity increasing from the poles to the equator, and with wet equatorial regions exhibiting the world’s highest levels of species richness (Hillebrand, 2004; Erwin, 2009). While the generality of the pattern among many groups of organisms suggests a common explanation, it is difficult to isolate specific causal components of latitude, such as solar radiation or temperature, from a multitude of factors that co-vary with latitude. Identifying factors that influence the diversification rate of a given clade are key to diversity hypotheses, with studies suggesting that the high tropical richness results from accelerated rates of diversification among many tropical clades (e.g., Cardillo et al., 2005; Ricklefs, 2006; Mittelbach et al., 2007; Wiens, 2007; Svenning et al., 2008). However, it is important to consider that low rates of extinction under benign and relatively stable climates rather than high rates of speciation alone may help explain contemporary species richness in the tropics.

On a global scale, distinctions have been made between contemporary processes that promote species richness and historical processes where past events and conditions have played a major role (Ricklefs, 1987; Jansson, 2003; Wiens and Donoghue, 2004; Cowling et al., 2014), For the former approach, correlations between plant species diversity and measures of water-energy availability at continental to global scales have been documented (O’Brien et al., 1998; Francis and Currie, 2003). However, it is not clear if such energy-richness relationships are causal because any variable that co-varies with latitude will tend to correlate closely with species richness on a continental scale (Jansson and Davies, 2008). Looking at geologic time-scales, it has been suggested that the observed diversity gradient may be a product of a latitudinal gradient in the amplitude of climatic shifts over time-scales of 10–100 kyr caused by Milankovitch oscillations in the Earth’s orbit and axial tilt (Dynesius and Jansson, 2000; Jansson and Dynesius, 2002). Rapid shifts in spatial patterns of climatic niches would be expected to drive high diversification rates (Moritz et al., 2000; Kozak and Wiens, 2010). It is notable that the Pleistocene was the coldest and most climatically unstable period in the Cenozoic, and that it has likely left a massive imprint on the distribution and abundance of species. By contrast, global hotspots of endemism have been linked to stable climatic refugia (Harrison and Noss, 2017).

**FIGURE 1:**
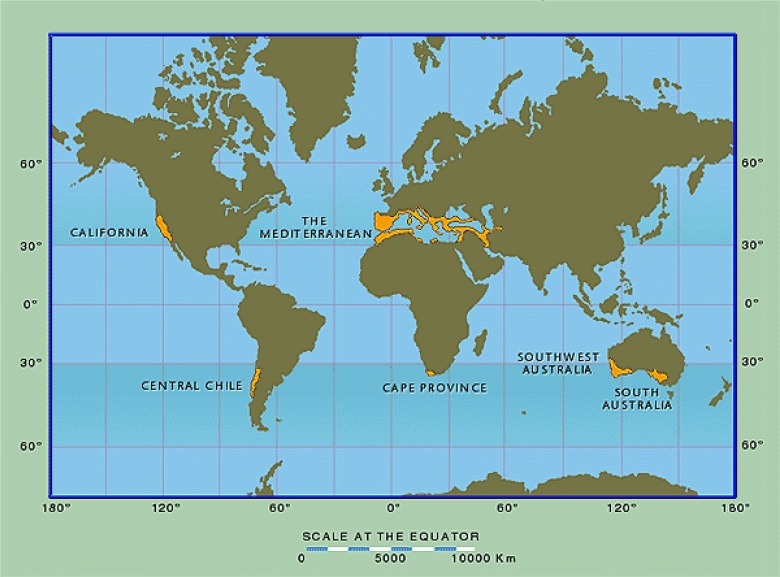
Global distribution of Mediterranean-climate regions. From Rundel et al. (2016); used with permission.

Missing in virtually every analysis of global patterns of biodiversity outlined above is any attempt to explain the remarkable levels of plant diversity present in the world’s five Mediterranean-type climate (MTC) regions, and most notably in the evergreen sclerophyll shrublands that dominate these regions, all of which are considered to represent biodiversity hotspots (Myers et al., 2000). These five regions include the Mediterranean Basin, California, central Chile, the Cape Region of South Africa, and southwestern Australia (**Figure 1**) and share the globally unusual climatic regime of mild wet winters and warm dry summers. Although collectively covering only about 2.2% of world land area, they are home to approximately one-sixth of the global flora (Cowling et al., 1996; Rundel et al., 2016). These five MTC regions are primarily positioned on the southwest margins of continental land masses at about 30–40^o^N and S latitude, confounding the classic pattern of reduction in species diversity with increasing latitude.

In this review we ask a series of questions. Are there environmental factors of climate and soils that can explain the species richness of MTC regions? How is fire regime related to the onset of MTC conditions in the Miocene? What plant life-history traits help allow for persistence in a fire-prone environment? What are the evolutionary implications of strategies of resprouters versus obligate seeders after fire? And finally, are MTC regions better considered museums of assembled biodiversity or instead nurseries of active speciation?

## Can Environmental Factors Explain Species Richness of the Mtc Regions?

There are several important considerations in developing hypotheses to explain the globally significant plant species richness of the five MTC regions. A number of ecological hypotheses have been suggested, with these typically centering on adaptation to seasonal drought, including adaptive radiation associated with heterogeneity of topography, climate, and soils (Hughes, 2017). In understanding species diversity, therefore, it is important to ask if there are ecological factors unique to MTCs that could explain their relatively high plant species diversities. Any hypothesis to explain the globally significant species diversity of the MTC regions must also take into account the differing levels of species richness between the regions across a wide range of spatial scales (Cowling et al., 2014). The Cape Region is consistently the most diverse of the five regions relative to area at any spatial scale, and arguably among the highest in the world per unit land area, followed by southwestern Australia as a distant second (**Table 1**). The diversity array scaled against area for both the western and eastern Mediterranean Basin show an intermediate pattern of contemporary plant diversity, followed by California and finally central Chile (Rundel et al., 2016).

**Table 1:** Plant species richness and fire-response traits in the five Mediterranean-type climate regions of the world.

	Mediterranean Basin	California	Cape Region	Southwestern Australia	Central Chile
**General characteristics**					
Area (10^6^ km^2^)	2.30	0.32	0.09	0.31	0.16
Winter precipitation (%)	75	90	75	72	90
Number of species (approx.)	25,000	4,700	9,000	8,000	2,900
Species/10,000 km^2^	2010	1377	3756	2261	1123
**Fire-related plant traits**					
Post-fire resprouting, woody species	+++	++	++	++	+++
Fire-stimulated germination	+++	++	+++	+++	+
- Heat-stimulated	+++	++	+++	+++	+
- Smoke-stimulated	++	++	++	+++	+
- Heat-tolerant seeds	+++	+++	+++	+++	++
Serotiny	+	+	++	+++	-
Pyrophytic annuals	+	++	-	-	-
Fire-stimulated flowering	++	++	++	++	+
Myrmecochory	++	+	+++	+++	+

*For fire-related plant traits, +++ = very widespread, ++ = present in diverse clades, + = rare, and - = trait is absent. Data on species diversity from Cowling et al. (2014) and species/10,000 km^2^ was predicted from the slope of species-area curves for each region rather than from dividing number of total species by area of region.*

The ancient and weathered landscapes of southwestern Australia and the Cape Region are characterized by highly acidic oligotrophic soils that are notably deficient in phosphorus, nitrogen and micronutrients (Lamont, 1995). These landscapes with their relative climatic and tectonic stability over the Cenozoic have a long history of floral diversification (Hopper, 2009; Cowling et al., 2014). Among ecological factors, it has been suggested that adaptation to oligotrophic soils involves a number of unusual mechanisms for coping with these edaphic conditions, and that this adaptive radiation has been associated with diversification (Lambers et al., 2010; Cacho and Strauss, 2014; Laliberté et al., 2015; Rajakaruna, 2017). One such mode of adaptation can be seen in the lineages that have evolved specialized root clusters that release carboxylates which solubilize inorganic phosphorus. This trait is best represented in species-rich lineages like the Proteaceae (Pausas and Lamont, 2018) but it is also present in other less diverse families (Groom and Lamont, 2015). The younger volcanic landscapes of the Chile have not been associated with extensive diversification of the Proteaceae despite the fact that all Chilean species exhibit cluster roots (Lambers et al., 2012), a legacy of their shared Gondwanan history. Both the Cape and southwestern Australia have high levels of edaphic endemism associated with granites, ferricretes, limestone and aeolinites (Cowling et al., 1994a; Hopper and Gioia, 2004; Byrne and Hopper, 2008; Lamont et al., 2016). Similarly, the California flora includes a diverse endemic flora associated with serpentine soils (Harrison et al., 2006). Most species in the Mediterranean Basin exhibit a clear segregation as adapted to limestone or acidic (granites, schists, etc.) soils. However, none of these geologies and associated soils is unique to the MTC regions and cannot alone account for the diversity patterns.

For the geologically younger and tectonically dynamic MTC regions of the Mediterranean Basin, California, and Chile, evolution across isolated habitat “islands” has been important in diversification (Rundel et al., 2016). In contrast to extensive diversification of sclerophyll shrubland lineages in the Paleogene to mid-Neogene in southwestern Australia and the Cape Region (Crisp et al., 2009; Rundel et al., 2016), speciation in these three regions suggests a relative absence of phylogenetic niche conservatism (Donoghue, 2008; Wiens et al., 2010), with a permeable habitat boundary open to recruitment of many lineages from adjacent biomes able to adapt to new and novel environmental conditions. For the Mediterranean Basin, this pattern of diversification can be seen in the complexity of topography, abundance of peninsulas and islands, edaphic conditions, and climate associated with narrow habitat ranges (Thompson, 2005; Médail and Diadema, 2009; Molina-Venegas et al., 2016), and speciation may be related to specific microhabitat conditions (Guzmán et al., 2009). In a similar manner for California, there are major gradients in topography, climate, and edaphic conditions, as well as geographic isolation as with the offshore California Islands. Although this complexity of habitat conditions has certainly promoted diversification, similar habitat complexity can be found in other temperate areas and thus is not unique to the MTC regions.

Chile also presents strong gradients in topographic and climatic conditions. However, its level of plant diversity, while impressive for the small size of the MTC area, is significantly lower than that of the Mediterranean Basin and California (**Table 1**; Cowling et al., 2014). With regard to the role of niche conservatism in the evolution of its flora, central Chile lies somewhere between the older MTC regions and northern hemisphere areas (Heibl and Renner, 2012; Jara-Arancio et al., 2014). Nevertheless, average elevation in central Chile is probably higher than in California and this might partially account for its overall lower diversity with respect to California (Arroyo, 2018). Even so, this factor is unlikely to totally explain what makes the Neogene evolution of the central Chilean flora so different from that of the other two tectonically younger and dynamic regions. One major difference between central Chile and all other MTCs is the minor role of fire as a selective force following the uplift of the Andes in the middle Miocene (Keeley et al., 2012; Rundel et al., 2016). This brings up the question as to what role might fire have played more generally in plant diversification in the other four MTC regions?

## Fire and the Onset of Mediterranean-Climate Conditions

Mediterranean-type climates with summer drought conditions are conducive to regular fire. The mild wet winter-spring seasons lead to moderate productivity generating broad landscapes of contiguous fuels, and the annual summer drought converts this biomass into available fuels (Keeley et al., 2012). Many parts of the globe are fire-prone, but among woody biomes MTC regions are perhaps unequaled in the seasonal predictability of fire, and this predictability contributes to selection for fire-adaptive traits (**Table 1**). In this regard, fire would seem to be important for our understanding of drivers of diversification in MTC ecosystems in view of increasing evidence that fire has been a major factor explaining global patterns of vegetation distribution and species diversity (Bond et al., 2005; He and Lamont, 2017; Pausas and Ribeiro, 2017). Conditions of seasonality became increasingly common in the Oligocene and early Miocene, and there is evidence for a global onset of MTC conditions of seasonality with summer drought with its relatively predictable crown fire regimes by 15–12 Ma (Rundel et al., 2016) and probably even earlier in some MTC regions (Lamont and He, 2017a). These conditions brought summer drought, a novel climatic condition with associated drought stress, but also importantly the onset of recurrent fire as a nearly ubiquitous component of selection in all of MTC regions, except central Chile. Prior to the Andean uplift in the middle Miocene, the present day strong east-west climatic differentiation across the relatively narrow southern South American land mass would have been far less differentiated with lightning strike fires likely during the summer months in central Chile. The significance of the simultaneous emergence of intensified fire compared with that of open woodlands and seasonal drought as a novel selective regime in four of the MTEs cannot be overemphasized (Lamont and He, 2017a).

At the time of first appearance of MTC regimes fire was not new to global terrestrial ecosystems (Bowman et al., 2009; Pausas and Keeley, 2009; Brown et al., 2012; Pausas and Ribeiro, 2013), but in the novel MTCs its widespread presence provided strong selection conditions for persistence in a fire-prone environment. In large part this was driven by the annual summer drought that made fire a highly predictable limiting factor, and thus a recurrent selective pressure, but one with mosaics of intensity in these ecosystems. MTC regions with their patterns of diversification in life-history and morphology related to this persistence in a fire-prone environment challenge the long held paradigm that plant diversity patterns may be largely explained by climate and soils alone (Raven and Axelrod, 1978; see Pausas and Lamont, 2018).

The role of selection for persistence in a fire-prone environment as a stimulus for diversification has received relatively little attention, due in part to the complexity of evolutionary mechanisms for fire-driven speciation (Cowling, 1987; Mucina and Wardell-Johnson, 2011; Schnitzler et al., 2011; Keeley et al., 2012; Ellis et al., 2014). Also underrated has been the significance of the diverse modes of adaptation to a particular fire regime (for example, see Pausas et al., 2018 on resprouting options among seed plants). Fire regime includes frequency, intensity, seasonality, and patterns of fuel consumption, with the potential for multiple temporal patterns of the fire regimes operating on the same landscape (Keeley et al., 2012). Paramount has been the need to show that fire-proneness preceded, or at least coincided with, the advent of novel fire-related traits (Lamont and He, 2017b).

Considered in the context of the intermediate disturbance theory (Huston, 2014), fire regimes offer a variety of ways that such impacts from fire can be expressed (Pausas and Ribeiro, 2013). Frequent disturbances with short intervals between fires select against species with a longer maturation time needed to reach reproductive maturity, or failure to resprout after fire (Lamont et al., 2013; Enright et al., 2014). At the other end of the disturbance gradient, a long interval between fires might exceed the lifespan of a plant species and not allow conditions for seed germination and seedling recruitment (Enright et al., 1998). Other axes of fire disturbance gradients such as fire intensity and fire seasonality can also influence selection and generate landscape mosaics promoting diversity and diversification in the longer term (Heelemann et al., 2008; Mucina and Wardell-Johnson, 2011).

**FIGURE 2:**
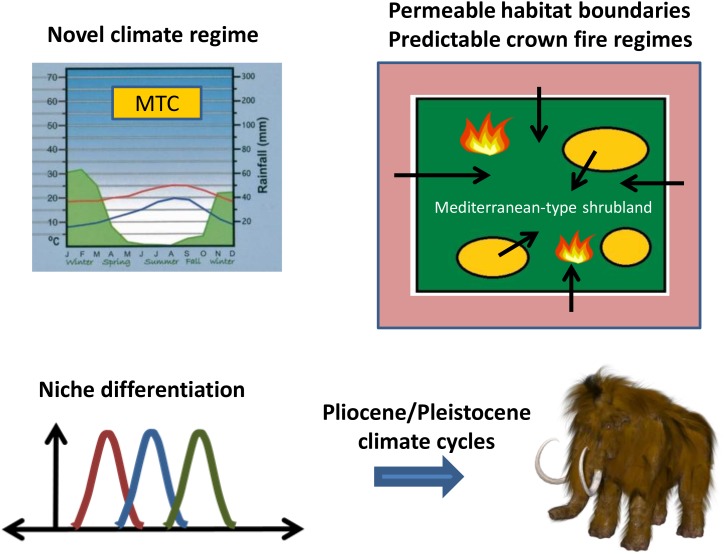
Evolutionary diversification of Mediterranean-region floras stimulated by the onset of a Mediterranean-type climate (MTC) with a crown fire regime in the Miocene, and enhanced by niche differentiation continuing through the Pliocene and Pleistocene. Niche drawing by Brews ohare; woolly mammoth by Max Pixel.

The expanding shrublands of MTC regions in the Miocene offered new habitats for colonization from a regional species pool for taxa with the ability to persist under novel conditions of climate, infertile soils, and intensified fire compared with those of open woodlands (**Figure 2**). Consistent with this persistence hypothesis is the breadth of independent lineages in disparate plant families that already possessed or developed key life-history strategies to cope with fire (Keeley et al., 2012; Lamont and He, 2017b). This pattern is in contrast to that present in the early Cenozoic lineages of southwestern Australia and the Cape Region (Crisp et al., 2009) and allowed for open recruitment from adjacent biomes. The assembly of a flora from a regional species pool across a permeable habitat boundary is parallel to that hypothesized for the diversification of the Brazilian cerrado from non-fire-prone ancestors in response to savanna expansion and a changing fire regime (Simon et al., 2009; Simon and Pennington, 2012). This contrasts with the Cape Region and southwestern Australia where the ancestors to the lineages diversifying in response to MTC environments were already fire-prone but with less intense fire (He et al., 2016; Lamont and He, 2017b).

## Persistence in a Fire-Prone Environment

Selection for persistence in response to fire as a key causal factor for species diversification in MTC regions has historically been under-appreciated or even ignored in its significance (Raven and Axelrod, 1978). However, the mechanisms for fire-driven speciation are widespread and may include both directional (novel traits) and stabilizing selection (retained traits) for adaptive morphological and life-history traits (Schnitzler et al., 2011; Keeley et al., 2012; Ellis et al., 2014; Enright et al., 2014; Lamont and He, 2017a). There is compelling evidence that fire has played a significant role in impacting the evolution of vascular plants during the Cenozoic (Lamont and He, 2017b). There is also strong evidence based on trait assignments to dated molecular phylogenies to show when fire became sufficiently extensive and recurrent to select for fire-related traits, in ancient lineages such as pines and *Banksia* (Proteaceae), confirming their fire-adapted status (Crisp et al., 2011; He et al., 2011, 2012). This evolutionary pressure can be seen in an array of adaptive traits in fire-prone environments that select for plant persistence. Thus, such traits may confer superior fitness, whether as adaptations or rarely as exaptations in the presence of a particular fire regime (Keeley et al., 2011; Mucina and Wardell-Johnson, 2011; Enright et al., 2014; but see Bradshaw et al., 2011 for a contrary view), and become genetically fixed through time (Paula et al., 2009; Lamont and He, 2017a). There are numerous examples of adaptive traits for fire persistence: (1) fire-resistant tissues in the form of thick bark and self-pruning of dead branches for trees; (2) high water content in succulent tissues; (3) belowground location of meristematic tissues followed by post-fire resprouting; (4) regeneration from soil-stored heat-resistant seeds; (5) serotiny with canopy seed storage and fire-stimulated seed release); and (6) fire-stimulated flowering (**Table 1**). Each of these forms of fire persistence is additionally influenced by the nature of the fire regime, in particular the frequency, seasonality, patch size, and intensity of fires (Mucina and Wardell-Johnson, 2011; Keeley et al., 2012). Some of these traits are discussed in the context of MTCs below.

Shrublands and heathlands dominate all MTC regions and have a crown-fire regime where intense fires sweep across broad landscapes scorching aboveground biomass. The dichotomy of resprouters and obligate seeders, whose parent plants are killed by fire, can be seen in many lineages of sclerophyllous shrubs in MTC regions (Pausas et al., 2004). Resprouters with fire-independent seedling recruitment survive fires as genetic individuals, replacing aboveground tissues structures by resprouting from insulated belowground meristems (Clarke et al., 2013; Pausas et al., 2018). In contrast, obligate seeders are killed by fire and their populations are reestablished by a new generation recruiting from soil- or canopy-stored seeds (Enright et al., 2007). Resprouting is promoted by conditions that do not favor recruitment or seed availability and is common when fires are exceptionally frequent, stochastic, or rare (Lamont et al., 2011; Cowling et al., 2018), while obligate seeding is promoted by moderate intervals of relatively high intensity fires within the lifespan of the species (Pausas and Keeley, 2014). Nevertheless, many fire regimes accommodate both fire-response types and their co-existence is common (Groeneveld et al., 2013; Vilagrosa et al., 2014). In central Chile, many woody species resprout well after anthropogenic fires (Donoso Zegers, 2006; Gómez-González et al., 2017), possibly reflecting an Oligocene or early Miocene history when their lineages would have been subject to more frequent fire before the uplift of the Andes.

One interesting dichotomy among obligate seeders lies in the formation of persistent soil banks versus canopy seed pools. A key difference between soil-storage and canopy-storage strategies is resilience to unpredictable fire cycles. Canopy seed storage becomes much less adaptive than soil-storage when the mean fire interval exceeds the lifespan of the parent plant (Lamont et al., 1991; Enright et al., 1998; Lamont and Enright, 2000; Keeley et al., 2012) or when it is shorter than the maturity age (Pausas and Keeley, 2014). Potential lifetime reproductive output is another important aspect of seed storage. Canopy seed storage is limited by determinant growth patterns that restrict lifetime seed accumulation to the available canopy space, whereas seed output is potentially much greater among species that produce seeds for soil-storage. At the generic level, canopy seed storage is common in southwestern Australia and the Cape Region, but uncommon in the Mediterranean Basin and California and virtually absent in central Chile. In central Chile, where serotiny has not developed, soil seed storage in woody plants is transient or short but is longer in some Fabaceae and Rhamnaceae with hard seed coats (Figueroa and Jaksic, 2004; Donoso Zegers, 2006; Gómez-González et al., 2017). Indeed, some evergreen tree species in the MTC area of Chile are known to have recalcitrant seeds (Donoso Zegers, 2006).

It has been hypothesized that the low-fertility soils of southwestern Australia and the Cape Region may have selected against soil-storage in favor of aerial storage because in such ecosystems high-nutrient seeds are subject to more intense predation when exposed on the soil surface (Keeley et al., 2011), or because the protracted time for maturation in the fruit enables limiting mineral nutrients to accumulate in the seed (Milberg and Lamont, 1997; Lamont and Groom, 2002). Alternatively, Cowling et al. (2005) showed that these two regions have the most reliable winter rains and that this is conducive to the evolution of on-plant seed storage and fire-stimulated seed release as this is more likely to lead to successful recruitment after all fires.

Obligate seeders with soil-storage disperse seeds in time as well as in space (Keeley et al., 2012) and thus most exhibit relatively unspecialized dispersal mechanisms. Capsules with numerous seeds unspecialized for dispersal are common in the five MTC regions. Exceptions are those species that exhibit myrmecochorous traits that confer a dispersal syndrome characterized by specialized seeds that attract ant dispersers. There is a pronounced difference in the degree of myrmecochory present in the five MTC regions (**Table 1**). The oligotrophic soils in the Cape fynbos and southwestern Australia kwongan are home to literally thousands of species that disperse seeds by ants (Bond and Slingsby, 1983; Lengyel et al., 2010). Although myrmecochorous traits are present in the other three MTC regions, these are rare and the total number of species in all of the other regions combined is an order of magnitude lower than in either fynbos or kwongan (Beattie and Hughes, 2002). The connection between myrmecochory and poor soils is inescapable, even within regions (Milewski and Bond, 1982; Cowling et al., 1994b), and provides a dramatic example of evolutionary convergence (Lengyel et al., 2010). Seed dispersal and burial in open sites reduces the incidence of granivory and incineration during the passage of fire (Auld and Bradstock, 1996), as well as disperses and buries seeds in open sites at a relatively inexpensive carbon cost of the “reward” (Keeley et al., 2012). Recent evidence from southwestern Australia indicates that vertebrates, such as emus and kangaroos, may also be attracted to the aril-bearing seeds and can transport them for several kilometers by contrast with the few meters by ants (Calviño-Cancela et al., 2008; He et al., 2009).

Beyond the iconic evergreen sclerophyllous shrublands and heathlands that form ecologically dominant assemblages across all five MTC regions, there are other woody and herbaceous growth forms that possess effective strategies of fire persistence. Geophytes are an important part of the post-fire flora in all MTC ecosystems, but are particularly prominent in the Cape Region and southwestern Australia. There is good reason to think of these taxa as presenting exaptations to fire as their primary response is to resprout seasonally from bulbs, corms and other underground parts (Keeley et al., 2012; Pausas et al., 2018), and this post-fire response is similar to their post-drought resprouting during the winter rainy season. Many geophytes in the MTC regions have fire-stimulated flowering. Some of these species are fine-tuned to only flower after fire and thus appear to be true fire adaptations (Lamont and Downes, 2011), but many other geophytes resprout and flower under a range of conditions, not only in response to fire. *Burchardia umbellata* (Colchicaceae), arising 11 million years ago, is of particular interest for not only does it have obligate pyrogenic flowering but it possesses MTC-restricted populations that have obligate summer dormancy (Lamont and He, 2017b).

Much of the species richness of the younger and dynamic landscapes of the Mediterranean Basin and California is formed by diverse clades of annual plants that have adapted well to the cool wet winters and dry summers. Many of these species have deeply dormant seeds with strict dependence on smoke to stimulate germination (Brown, 1993; Dixon et al., 1995; Keeley and Bond, 1997). There is good evidence that this trait evolved through convergence across several of the MTC regions (Keeley and Pausas, 2018). Annuals are less important in central Chile compared with California (Arroyo et al., 1995) and have largely evolved without the influence of fire.

In central Chile, some native herbaceous species clades have the potential to form persistent seed banks with annual species capable of persisting in the soil for longer than perennial species (Arroyo et al., 2006). The existence of an abundant but fairly short-lived herbaceous soil seed bank is typical (Figueroa and Jaksic, 2004; Gómez-González et al., 2011a). Native annual richness and abundance, with some exceptions, tends to be depressed by high- but not by low-intensity litter burning (Gómez-González and Cavieres, 2009; Gómez-González et al., 2011b). Smoke- and heat-stimulated seed germination occurs in some herbaceous species in central Chile (Figueroa et al., 2009; Gómez-González et al., 2011b; Figueroa and Cavieres, 2012), but the majority of species are fire-intolerant, with an increasing number negatively affected as fires become more intense (Gómez-González and Cavieres, 2009). Smoke- and heat-stimulated germination are present in less than a quarter and one-third of woody species in central Chile, respectively, but are mostly limited to colonizers (Muñoz and Fuentes, 1989; Gómez-González et al., 2008, 2017); however, close to half of all woody species are negatively affected and heat tolerance is far less evident than in herbaceous species (see also **Table 1** for a comparison of the incidence of fire-response traits for central Chile and among MTC shrublands).

## Evolutionary Implications for Resprouters and Obligate Seeders

Because obligate seeders often have shorter generation times than resprouters, this life-history trait could impact on the evolution of these adaptive strategies at both microevolutionary (Verdú and Pausas, 2007; Segarra-Moragues and Ojeda, 2010) and macroevolutionary scales (Bond and Midgley, 2001; He et al., 2011, 2012). Indeed, generation time has been shown to be negatively correlated with molecular substitution rates in angiosperms (Smith and Donoghue, 2008), allowing shorter-lived species to explore a wider environmental space than longer-lived species (Smith and Beaulieu, 2009). This may be one of the reasons for a higher proportion of annual species in the three tectonically active MTC regions than in non-MTC temperate shrublands. It may be significant that the proportion of annual species in the central Chilean flora is much lower than in California (Arroyo et al., 1995). However, there are also potential adaptive advantages associated with resprouters as repositories of past genetic innovations. Obligate seeding species subject their gene pool to intense selection because each fire results in a new generation. Thus, the characteristics of individuals that are selected by circumstances associated with one fire regime may not be subsequently selected if the environment changes, with the rate of change significant.

When Wells (1969) drew attention to the dichotomy of obligate seeders and resprouters, he argued that the obligate seeding strategy leads to much greater rates of speciation. Attempts have been made to understand the relative diversification rates of obligate seeders vs. resprouters (Bond and Midgley, 2001; Lamont and Wiens, 2003; Verdú and Pausas, 2007; Boatwright et al., 2008; Litsios et al., 2014). However, the results are inconclusive because in many of these comparisons the resprouters were also facultative seeders, and thus did not represent a good test of obligate seeding vs. obligate resprouting (Keeley et al., 2012) and because of confounding by the high evolutionary lability between the two fire-response types (He et al., 2011). When studying the whole community rather than a single lineage, there is evidence for the Mediterranean Basin flora of higher speciation rates of lineages associated with the seeder life-history than those lineages associated with obligate resprouting (Verdú and Pausas, 2013; Vargas et al., 2018).

Recent studies of the Restionaceae, an ecologically important group in oligotrophic heathlands of the southern regions of the African and Australian continents (Linder, 2003; Linder et al., 2003), provide an interesting insight into this question. Obligate seeders have diversified faster than resprouters in the Cape Region, but not in southwestern Australia, possibly reflecting a difference in the landscape heterogeneity of the two regions (Litsios et al., 2014). Complicating any simple conclusion, however, is the history of greater fire activity in southwestern Australia extending over 100 million years (Lamont et al., 1985; He et al., 2016). Speciation has hypothetically proceeded more rapidly in the obligate seeder lineages of the Cape Region because of the topographic and climatic mosaics across the landscape. However, collation of many adaptive traits for 40 genera in the flora of the Mediterranean Basin has shown that the diversification of seeders there can be attributed to fire-stimulated germination of their soil-stored seeds and that altitudinal variation is irrelevant (López-Villalta, 2014). Rapid shifts between the fire-response traits of obligate seeding and resprouting rather than differential amounts of extinction counterbalance this greater rate of speciation, resulting in a relatively even balance between the two fire-response traits, except for the Cape Region where obligate seeders have speciated more profusely than resprouters in most endemic clades (Keeley et al., 2012).

## Museums or Nurseries?

Timing of diversification is crucial to understand the origin and evolution of plant diversity in any biodiversity hotspot such as the five MTC regions. Paleobotanical and phylogenetic evidence help elucidate whether accumulation of either ‘ancient clades’ (museum hypothesis) or ‘new differentiation’ (nursery hypothesis) better account for the levels of diversity observed (Rabosky and Hurlbert, 2015). In other words, can the plant diversity of the five MTC regions today be explained by a museum hypothesis of accumulated ancient lineages or better by a nursery hypothesis with rapid evolutionary diversification of young lineages influenced by fire? The answer to this question is complex. There are two main components to consider: phylogenetic differentiation and the evolution or adoption of morphological and life-history traits involved in persistence in a fire-prone environment. For a given species richness the museum hypothesis can produce higher phylogenetic diversity than the nursery hypothesis if numerous paleo-endemic elements are present. A mutually non-exclusive hypothesis—i.e., occurrence of spatio-ecological limits—has to be considered to evaluate whether all MTC regions have reached their limit in carrying capacity because of ecological and spatial constraints to diversity (Rabosky and Hurlbert, 2015).

Consistent with the museum hypothesis, a number of species-rich lineages in southwestern Australia and the Cape Region, most notably in the Proteaceae, Myrtaceae and Haemodoraceae, have ancient origins that proceeded under the influence of fire well before the development of MTCs (Crisp et al., 2011; He et al., 2016; Lamont and He, 2017b). Gradual diversification in fire-prone environments through the Cenozoic, associated with low rates of extinction, has had an impact in influencing the assembly of modern levels of species richness for many lineages in these two regions (Verboom et al., 2009; Cowling et al., 2014). Nevertheless there have been high rates of diversification in many lineages since the onset of a MTC in the Miocene. It is interesting to compare patterns of diversity in fynbos clades between the western Cape with a classic MTC regime and the eastern Cape with aseasonal rainfall. Species diversification in fynbos clades has been much higher in the former (Forest et al., 2018).

In contrast, the climatically unstable Mediterranean Basin has offered fewer opportunities for diversity accumulation in the long term, but it is a hotspot of recent rapid speciation (Valente and Vargas, 2013). Nevertheless, Mediterranean lineages with species favored by the presence of fire include, on the one hand, ancient lineages such as the single species of the only taxonomic family (Drosophyllaceae) endemic to the Mediterranean Basin, and on the other hand, young lineages such as the monotypic genus *Pseudomisopates* (Plantaginaceae) (Gómez-González et al., 2018; Vargas et al., 2018). Parallel examples of old, poorly diversified woody lineages can be seen in central Chile (Rundel et al., 2016).

There are prominent paleo-endemic lineages of woody plants in all five MTC regions whose ancestry dates back to the Oligocene or earlier. Examples for California include a number of isolated chaparral lineages such as *Pickeringia* (Fabaceae), *Adenostoma* (Rosaceae), *Cneoridium* (Rutaceae), *Malosma* (Anacardiaceae), and *Bergerocactus* (Cactaceae) (Baldwin, 2014), that are well adapted to their fire-prone environment. Although a few of these lineages remain ecologically significant today, they have not diversified and contribute little to overall floristic richness at the species level (Lancaster and Kay, 2013). However, these paleo-endemics do provide a contribution to the phylogenetic diversity of each region.

To assess the nursery hypothesis, it is necessary to ask if rates of diversification today and in the past have been unusually high in the MTC regions and how fire regimes may have influenced this diversification (**Figure 2**). Despite the overall species richness of these regions, rates of net species diversification for most lineages are generally not remarkably high, at least in comparison with species-rich regions such as the *páramo* (Madriñán et al., 2013). Although a few individual MTC lineages have exhibited extremely high rates of Neogene diversification, as exemplified by *Dianthus* in the Mediterranean Basin (Valente et al., 2010a,b) and core Ruschioideae (Aizoaceae) in the Cape Region (Klak et al., 2004), richness cannot be explained overall by high diversification rates in the MTC regions as a whole. In the same manner, phylogenetic studies on lineages shared between MTC regions and elsewhere indicate that contrasting diversification rates do not explain differences in extant species richness (Hopper, 2009; Sauquet et al., 2009; Valente et al., 2011; Buerki et al., 2012; Valente and Vargas, 2013). The high diversity of *Protea* in the Cape Region, for example, has occurred because the clade has been in the Cape almost three times longer than in the adjacent savanna grasslands (Lamont et al., 2017). However, a diversification study at the community level for the Mediterranean Basin showed increased speciation with the hypothesized onset of the MTC and fire (Verdú and Pausas, 2013). Consistent with this is the recent study of 27 dated clades in the Mediterranean Basin showing that species-rich clades (with more species and higher diversification rates) tended to diversify later (post Miocene) than species poor clades (Vargas et al., 2018). Phylogenetic analyses of new comparative studies on diversification rates in subtropical and other temperate regions would be welcome.

Spatio-ecological limits to diversification have been explored in the Cape Region and Mediterranean Basin. An indirect way of investigating the existence of diversity limits is by testing the relationship between clade age and species diversity. If the diversity in a region depends significantly on the diversity carrying capacity/ecological limits for that area, then there should be no relationship between clade age and diversity (Rabosky and Hurlbert, 2015). There is no such relationship for plant clades of the Mediterranean Basin (Valente and Vargas, 2013). In contrast, there is a complex relationship between clade age and richness in the Cape Region. There is evidence of saturation for some clades in the mountains, but in the lowlands, where there have been many more ecological opportunities for speciation since the Miocene; many clades are still radiating rapidly and do not appear to have reached a diversity limit (Valente and Vargas, 2013; Cowling et al., 2017). The same approach has been applied for some lineages of the southwestern Australia flora, where indications of limits to ecological diversity were found (Cook et al., 2015). Spatio-ecological limits need to be tested for the other MTC regions and for lineages including species adapted to fire that may or may not have reached diversity carrying capacity (i.e., a diversity limit).

Both the museum and nursery hypotheses have important relevance in explaining species richness of the MTC floras, with fire as a strong stimulant for diversification in a manner not present in other temperate ecosystems where fire seasonality and regimes differ (Murphy et al., 2013). The Mediterranean Basin, California, and central Chile are seen as nurseries for strong rates of Neogene diversification, while the older landscapes of southwestern Australia and the Cape Region show significant components of both Paleogene and younger Neogene speciation in their diversity. Low rates of extinction suggesting a long association with fire more than high rates of speciation have been key to the extant levels of species richness.

## Author Contributions

The authors represent a collaborative group of colleagues who jointly conceived, designed the research, and carried out the project. PR took the lead on writing and all coauthors participated in editing resulting drafts.

## Conflict of Interest Statement

The authors declare that the research was conducted in the absence of any commercial or financial relationships that could be construed as a potential conflict of interest.
